# Research on the impact of sports event image on tourism loyalty in heritage sites: An empirical study based on the Yongding Tulou marathon

**DOI:** 10.1371/journal.pone.0326333

**Published:** 2025-06-17

**Authors:** Juan Du, Guifeng Zheng, Jingxuan Liang, Yongqiang Ma

**Affiliations:** 1 Department of Public Physical Education, Fujian Agriculture and Forestry University, Fuzhou, China; 2 Physical Education Department, Xiamen University Tan Kah Kee College, Zhangzhou, China; 3 College of Humanities & Social Development, Nanjing Agricultural University, Nanjing, China; 4 Anxi College of Tea Science, Fujian Agriculture and Forestry University, Quanzhou, China; University of Naples Federico II: Universita degli Studi di Napoli Federico II, ITALY

## Abstract

Heritage tourism related to sports events is widely recognized as a new paradigm of integration between culture, sports, and tourism. By combining sports events with world heritage sites, it attracts more tourists to pay attention to world heritage, serving as an important practice for the protection and sustainable development of world cultural heritage. However, the academic community still lacks discussions on its composition and impact effects. This study, based on the event of the 2022 Yongding Tulou Marathon in Fujian, constructs a multiple impact model of sports event events on tourist loyalty to heritage sites, integrating social and market perspectives. It follows the logic of “recognition-emotion-behavior” and uses PLS-SEM for empirical testing. PLS-MGA is conducted based on tourist demographic characteristics to explore the impact of different consumer group characteristics on the influencing paths. The results show: (1) The image of sports events is an important factor affecting tourist loyalty and the image of heritage sites, but it is difficult to directly influence tourists’ sense of place attachment; (2) The image of heritage is more deeply rooted in the heart, prompting tourists to develop loyalty and attachment to the heritage site, while attachment can also directly stimulate tourist loyalty; (3) The image of sports events can enhance the cognitive image of heritage through positive tourist experiences, thereby improving tourist loyalty to the heritage site. Additionally, the image of sports events can also stimulate tourists’ heritage image and sense of place attachment, ultimately strengthening loyalty to the heritage site; (4) Demographic variables such as gender, age, frequency of participation, and monthly running distance are key factors affecting the mechanism through which the image of sports events influences destination loyalty. The research findings not only reveal the theoretical process of how the image of event events influences multiple behaviors of tourists at heritage sites but also provide a comprehensive perspective and methodological tools for the study of sustainable development of world heritage sites. They also offer theoretical references for managers to effectively leverage the positive effects of developing sports tourism projects in heritage tourism destinations.

## 1. Introduction

The sports tourism industry is an emerging industry centered on the sports industry and carried by the tourism industry. It is a product of the deep integration of sports and tourism in the current era [1]. Currently, the global sports tourism industry is mainly carried out in the form of ice and snow sports tourism, coastal sports tourism, and sports event tourism. Among them, sports event tourism is a new form that combines sports events with tourism. With the upgrading of sports, culture, and tourism consumption and the deep integration of industries, “following events to travel” has gradually become a new popular leisure direction for the public. The form of promoting “tourism” through “competition” and “culture” has gradually become the mainstream of the times. In addition, the emergence of sports event tourism has broken the traditional closed competition format. Through the tourism experience before and after the competition, it effectively promotes the physical and mental relaxation of participants and plays an important role in deepening urban construction, driving tourism consumption, and promoting regional economic development.

Marathon event tourism is an important component of sports event tourism. According to the Blue Paper on China’s Road Running Events 2023 released by the Chinese Athletics Association, in 2023, China alone hosted 699 road running events, with the number of marathon events (including half marathons) accounting for more than 90% and the total number of marathon participants approaching 5.5 million. The increasing growth of sports tourism and its expanding social influence have been regarded as one of the important indicators of urban development [2]. At the same time, driven by the integration of culture and tourism [3], World Heritage Sites (WHS) embody rich historical and cultural value and unique natural landscapes, providing distinctive venues for event tourism. With their profound cultural heritage, abundant architectural remains, and beautiful natural environments, these sites can bestow unique atmospheres and significance on events, thereby creating more distinctive tourist experiences. Therefore, WHS are increasingly being utilized as sports tourism resources, particularly the widespread sporting events held at heritage sites, which attract participants from around the world [4,5]. The promotion of marathon activities at World Heritage Sites is becoming an important choice for diversified tourism activities today. For example, events such as the Yongding Tulou International Marathon in China, the Himeji Castle Marathon in Japan, the Halong Bay World Heritage Marathon in Vietnam, the Jerusalem Marathon, and the UNESCO City Marathon integrate heritage tourism experiences with international marathons. As two core areas of the modern cultural and tourism industry, the significance of heritage tourism and sports events is not only reflected in the economic benefits, but also involves the reconstruction of cultural identity, the accumulation of social capital and the realization of sustainable development goals. Through the synergy effect of heritage tourism and sports events, holding sports events in heritage sites can create the experience of “modern practice in the historical context” [6], achieving a breakthrough reconstruction of spatio-temporal narratives and a deep activation of emotional connections. These events allow participants to fully experience the cultural journeys of world heritage and are gradually becoming a new trend in event tourism, thereby promoting industrial transformation and upgrading [7].

Existing research on sports tourism has already yielded a relatively rich body of theoretical achievements. The research frontier primarily revolves around the performance of large-scale events [8], influencing factors [9], management, and resident support [10,11] With the popularization of the internet, research topics such as sustainable development [12], small-scale events [13], and online sports event consumption [14] have begun to emerge. Additionally, visitor behavior represented by loyalty and satisfaction remains a key focus in the field of sports tourism today [15,16], and is crucial for evaluating the effectiveness of tourism marketing and promoting the sustainable development of the tourism industry [17]. As a new model of current tourism marketing, the combination of heritage tourism and sports tourism has seen many practical attempts [18]. However, there is a lack of systematic theoretical exploration. No scholars have yet revealed the impact of sports events and heritage characteristics on visitor loyalty and behavior from a theoretical perspective, and the relationship between these two factors remains unclear. Moreover, individual characteristics of visitors are also an important factor influencing their behavior [19]. In the context of sports tourism, how individual characteristics of visitors at heritage sites affect their behavior remains to be further discussed. Therefore, to address these research gaps, this study will explore the impact of sports events on visitor loyalty at heritage sites from the perspective of sports tourism, aiming to address the following three issues: (1) How can sports tourism events and heritage images better stimulate visitor loyalty? (2) Is there a mediating mechanism? (3) Can demographic characteristics influence this mechanism?

In summary, this study intends to use PLS-SEM analysis to explore the impact mechanism of sports tourism events on the formation of visitor loyalty at heritage sites. In the hope of innovating through mechanism deconstruction: breaking through the single path of “motivation - behavior” in traditional tourist loyalty research, based on the perspective of cognitive-emotional integration, reveal the chain transmission path of the image of sports events through the cognitive reshaping of sports events → emotional generation → strengthening of tourist loyalty, and provide a dynamic interpretation framework for understanding “event-driven heritage tourism”. Furthermore, the PLS-MGA method was employed to verify the heterogeneity of the moderating effects of different demographic variables in the image transmission path, with the aim of proposing the optimal marketing strategy for the combination of sports tourism and heritage tourism. This is intended to provide feasible references for the rational development of sports events participating in the protection of world heritage, and promote the high-quality development of the tourism industry.

## 2. Literature review

### 2.1. Sport event image and heritage image

The concept of image originated in the retail industry and was initially applied in marketing-related fields in the early 1990s [20]. Subsequently, it has been extended to the field of sports tourism. Sporting events inherently possess competitive, skill-related, and social characteristics. Event image in the minds of sports tourists may encompass aspects such as organization, competition, environment, social interactions, sense of achievement, and emotional involvement in the event [21,22]. The event image is defined as the “accumulated interpretation of consumers’ meaning or associations with the event” [23]. Additionally, the event image is related to its functions (i.g. sports, festivals, and arts), characteristics (i.g. scale and professional levels), and individual aspects. Through existing infrastructure, organizing sports events can create long-term image benefits [24]. On the other hand, heritage image is an overall impression formed by the integration of natural landscape, historical culture and cultural atmosphere, which will constantly evolve with the changes of time, tourism development, heritage protection measures and other factors. These specific attributes together form a coherent and complete image of cultural heritage. It is associated with the current image in the construction of cultural heritage along with the passage of time [25]. The features of the games and cultural heritage can enhance the tourist experience together [26]. Especially for marathon participants, they are more inclined to choose unique places to participate [27], in order to obtain different race experience. The literature on the image of the games and the image of the heritage further shows that the cultural heritage sites attract many participants by their own unique charm [25]. Thus, the following hypotheses of the study arise:

*H1: Sport Event image has a significant positive impact on the heritage image.*

### 2.2. Mediating role of heritage image

Numerous studies have shown that destination loyalty is often conceptualized and measured to include intentions for revisiting and recommending the destination [28–30]. Therefore, this study argues that loyalty consists of tourists’ word-of-mouth intends to positively recommend others to visit the heritage marathon destination and positive behavioral intends to re-engage in the future with the heritage marathon or other activities conducted at the destination.The event image can impact tourists’ behaviors, such as their willingness to participate in events again or revisit the destination [31–33]. From the perspective of cognitive psychology, the analysis of the influence mechanism of marathon events on the loyalty of tourists in heritage sites is a dynamic process of “cognitive-emotion-behavior” [34]. The experience of tourists in competitions can affect their attitude towards the products or services of tourism destinations [19]. When visitors receive good event sports, they will develop more attention and interest in such events and the places where they are held. This makes it easier for visitors to remember the experience and prioritize it when they choose to participate in the marathon in the future. Scholars have pointed out a direct correlation between sports tourists’ event experiences and destination loyalty [35–37]. As stated above, the event image could influence tourists’ attitudes and behaviors at heritage tourism destinations. Therefore, this study posits that the event image contributes to enhancing destination loyalty.

At the same time, research on heritage image and tourist loyalty points out that tourists with a positive heritage image are more likely to form positive impressions of their experience [25,38]. It can strengthen tourists’awareness and emotional connection to the destination, leading to tourism behaviors such as loyalty and recommendations [39,40]. Zuo pointed out that elements like authenticity in the heritage image can stimulate tourist behavior, supporting the accuracy of this view [41]. In addition, according to the theory of “cognitive-emotion-ideation” [34], the characteristics of the games and cultural heritage can jointly enhance tourists’ travel loyalty and other behaviors [26]. It is evident that the image of sports events can profoundly influence visitors’perceptions of heritage sites, establishing a positive heritage image.A good heritage image can encourage behaviors such as promotion and protection among tourists. Therefore, this study posits that the image of sports events can affect tourists’heritage image, thereby fostering loyalty to the heritage site and a greater willingness to engage in tourism activities at that site, suggesting the existence of a mediating relationship. Based on the above, this study proposes the following hypotheses:

*H2: The sport event image directly significantly, and positively influences tourists’ destination loyalty*

*H3: Heritage image significantly positively influences tourists’ destination loyalty.*

*H4: Heritage image mediates the relationship between sport event image and destination loyalty.*

### 2.3. Mediating role of place attachment

Scholars define place attachment as the cognitive/emotional connection people have with a place, which is formed through a combination of emotions, sentiments, knowledge, beliefs, and behaviors related to that place [17]. Prior research has shown that a strong relationship between event participants and the event location can elicit powerful reactions towards the destination, which enhances positive outcomes such as participants’ destination loyalty [42]. Regular sports events can enhance the attachment of sports visitors to the host place. In the process of participating in the competition, sports tourists have in-depth experience in the heritage sites, have emotional resonance with them, extend their preference for the games to the host place, and then develop attachment to the host place.

At the same time, according to the “cognitive-emotion-behavior” theory of cognitive psychology [34], Sufficient evidence exists indicated that attachment and image are components of attitude, and attachment is regarded as the emotional component of attitude, while image is more inclined to the cognitive component of attitude [31,43]. in the process of experiencing tourism, tourists at heritage sites will be influenced by the external marathon experience and the image of cultural heritage to generate individual emotional identity [44]. Meanwhile, studies advocate for the examination of place attachment as a mediating factor in the relationship involving destination loyalty [45]. Furthermore, empirical evidence demonstrates that place attachment influences subsequent destination loyalty [46,47]. From the above analysis, it is evident that the image of sports events and heritage image can foster visitors’attachment to heritage sites, and this attachment serves as a motivation for tourists to revisit, and inspire loyalty from visitors. Therefore, the study posits that the image of sports events can influence tourists’place attachment, thereby fostering loyalty to the heritage site. Additionally, the image of sports events can also affect the heritage image, leading to increased attachment and ultimately resulting in visitor loyalty. In summary, the following hypotheses are proposed in this study:

*H5: The sport event image has a direct and significantly positive impact on place attachment.*

*H6: The heritage image has a direct and significantly positive impact on place attachment.*

*H7: Place attachment has a significantly positive impact on destination loyalty.*

*H8: Place attachment mediates the relationship between sport event image and destination loyalty.*

*H9: Heritage image and place attachment play a chain-mediating role in the relationship between sport event image and destination loyalty*

### 2.4. Multi-group testing

Different demographic characteristics, such as gender and age, can influence tourists’ behaviors, leading to varying emotions and actions toward the same tourism product. Research has demonstrated that demographic factors like age and culture are significant determinants of behavioral tendencies [48]. By conducting a multi-group analysis, differences between various groups can be considered, allowing for the development of targeted and precise marketing strategies [49,50]. In summary, based on the verification of model relationships using overall data, this study employs multi-group analysis with gender, age, and income as moderating variables among demographic factors, and proposes the following hypotheses.

*H10: The relationships among all variables in the model exhibit significant differences across tourist groups with different genders, ages, participation frequencies, and monthly travel volumes.*

**Fig 1:**
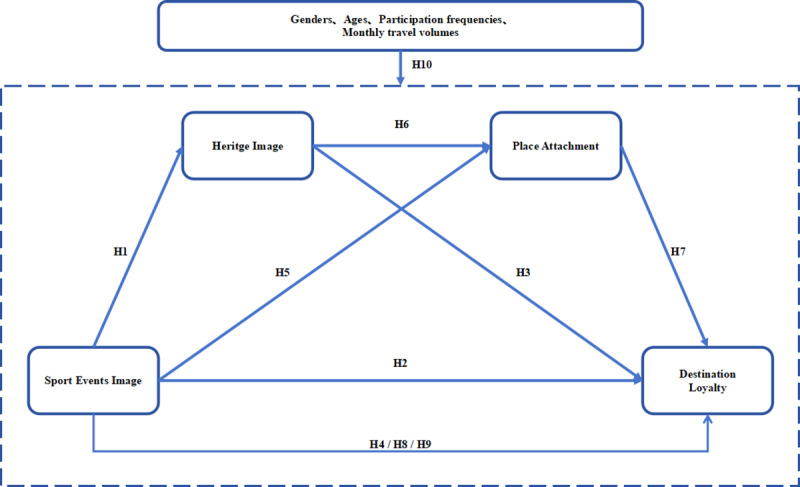
Conceptual Model.

Overall, the study, grounded in image transfer theory and attachment theory, provides psychological explanations for tourists’ destination loyalty in hosting sports events under the influence of cultural heritage. Employing theory to enhance comprehension of tourists’ consumption behavior, the theoretical model constructed by the study is shown in Fig 1, derived from the preceding analysis and hypothesis deduction.

## 3. Research design

### 3.1. Case study overview

Yongding Tulou, located in Longyan City, Fujian Province, on the southeast coast of China. It is the core area and birthplace of the Fujian Tulou, a UNESCO World Heritage site. Among them, the “King of Tulou,” Chengqi Lou, and the “Prince of Tulou,” Zhencheng Lou, are particularly noteworthy and admired worldwide. The Yongding Hakka Tulou is renowned for its long history, diverse styles, grand scale, ingenious structure, complete functionality, and rich cultural significance. As of now, Yongding preserves over 30 types of Tulou, including round, square, pentagonal, octagonal, and hat-shaped ones, totaling more than 22,000 buildings.Yongding Tulou Marathon has been held regularly each year since 2016. The entire event experience connects human history, natural scenery, and the image of the World Heritage, showcasing the image of Yongding Tulou Hakka culture and the World Heritage. Hence, the study selects the Yongding Tulou Marathon as a case study to explore mechanisms that contribute to promoting tourist destination loyalty and benefiting the inheritance and protection of the world heritage of Yongding Tulou.

### 3.2. Measurements

Most of the scales selected for this study are relatively mature international English scales, and the biggest problem encounted when translating foreign languages scales or tests is cross-cultural differences. Therefore, scholars addressing cross-cultural considerations when translating research tools often adopt a back-translation approach. Firstly, the study invited ‌two English teachers from the Foreign Languages Department of Fujian Agriculture and‌ to Forestry University translate the English scales into Chinese using the back-translation procedure. Secondly, the study invited ‌three experts in the field of heritage tourism‌ from the Tourism College of Huaqiao University, the Foreign Languages and Tourism College of Liming Vocational University, and the Anxi Tea College of Fujian Agriculture and Forestry University to evaluate the translated scales. Finally, the study made appropriate modifications based on the actual scenarios of sports tourism and heritage tourism. Among them, Heritage Image draws on the relevant research of Wu & Li [51], while sport events Image draws on Huang et al. [52], place attachment draws on the relevant studies of Ramkissoon et al. [43], destination loyalty drew reference from the relevant research of Zhang et al. [53]. All scales were measured using a 7-point Likert scale. The final scale was translated back to English by two English teachers.

### 3.3. Data collection and sample descriptive statistics

The study was conducted under the review and approval of the the Anxi College of Tea Science, Fujian Agriculture and Forestry University (NO. FAFUaxcxy-2022-06).Before the formal survey, a pre-survey was conducted. Based on the list of participants in previous Yongding Tulou Marathon events provided by the organizers, 100 people were randomly selected for an online questionnaire survey, and 86 valid questionnaires were recovered. The questionnaire was subjected to reliability and validity tests, revealing that item numbers 2, 3, 5, 9, and 10 for event image, item NO.3 for destination loyalty, and item NO.4 for place attachment did not meet the minimum requirement of a factor loading of 0.40 or had cross-factor loading problems. Therefore, these problematic items were excluded from the questionnaire.

According to official reports, the total number of participants in the 2022 Fujian Yongding Tulou Marathon is only 3,500, including 1,000 for the full marathon, 1,000 for the half marathon and 1,500 for the tulou style run (5 km). The main survey targeted participants who attended the Yongding Tulou Marathon on December 25, 2022. The survey period is from 20/12/2022 to 30/12/2022. The survey objects of the formal questionnaire in this study were participants in the 2022 Fujian Yongding Tulou Marathon. The questionnaire was distributed and collected by offline random sampling method. Before the questionnaire was distributed, members of the research team would make a brief introduction to the interviewees and distribute the questionnaire to the tourists after asking for their consent. Unfortunately, due to the limiting factors of the Covid-19 pandemic at that time, the questionnaire delivery needed to be filled in face-to-face. When the research team members asked for the questionnaire delivery, many participants did not agree to fill in the questionnaire due to their own health concerns. Therefore, in the process of distributing questionnaires, the team tries to distribute as many questionnaires as possible. In the end, for out-of-town participants, 305 questionnaires were distributed through face-to-face surveys, and 278 valid questionnaires with an effective recovery rate of 91.1%. The specific distribution of samples was that there were mostly male participants, i.e.,183 participants, accounting for 65.8%, and aged 36–45, totaling 90 participants, accounting for 32.4%. There were 171 participants from other regions of Fujian Province, accounting for 61.5%. Regarding marathon experience, the majority participated once, with 161 people, representing 57.9%. Runners with a monthly running distance of 50–100 kilometers (ordinary novice level) totaled 73 people, accounting for 26.3%. The majority of marathon participants ran in pairs, with 207 people, representing 74.5%. Among the runners, 174 people, or 62.6%, found the cultural heritage deeply impressive during the entire race experience. The length of stay of tourists was mostly 2 days, with a total of 194 people, representing 69.8%. Leisure and entertainment expenditures before and after the marathon were mostly 201–300 yuan and 500 yuan or more, with 59 and 57 people respectively, accounting for 21.2% and 20.5% (Table 1).

**Table 1:** Sample situation.

Table 1 Sample descriptive statisticsSample characteristics.	Sort	Number of samples/copies	Proportion/%
Sex	Male	183	65.8
	aFemale	95	34.2
Age	18-25	40	14.4
	26-35	54	19.4
	36-45	90	32.4
	46-55	72	25.9
	Over 55	22	7.9
Place of residence	Longyan	91	32.7
	Other cities in Fujian	171	61.5
	Other cities in China	15	5.4
	Beyond the borders	1	0.4
Number of participation	1	161	57.9
	2	88	31.7
	3	19	6.8
	4	10	3.6
Monthly mileage	<20 km	53	19.1
	20-50 km	72	25.9
	50-100 km	73	26.3
	100-200 km	58	20.9
	>200 km	22	7.9
Group running	Yes	207	74.5
	No	71	25.5
Impressive event	Racing club	81	29.1
	Destination	23	8.3
	Cultural heritage	174	62.6
Length of stay	1 day	61	21.9
	2 days	194	69.8
	3 days	23	8.3
Entertainment expenses	0-100 yuan	38	13.7
	101-200 yuan	47	16.9
	201-300 yuan	59	21.2
	301-400 yuan	44	15.8
	401-500 yuan	33	11.9
	Over 500 yuan	57	20.5

## 4. Results

### 4.1. Goodness of fit

In contrast to CB-SEM, which places significant emphasis on discussing the model’s goodness of fit, PLS-SEM typically involves less discussion on model fit [54]. However, this does not imply that goodness of fit testing is irrelevant in PLS-SEM. Scholars such as Henseler have introduced the Standardized Root Mean Square Residual (SRMR) as a goodness of fit criterion for PLS-SEM, which provides a better interpretation of these models and can help avoid model misspecification. When SRMR is less than 0.08, scholars consider it to indicate a good fit [55]. Through calculations using SmartPLS, the SRMR value for this study was found to be 0.049, indicating that the structural model in this study has a good fit.

### 4.2. Reliability and validity

The data collected were processed using Smart PLS to assess the reliability and validity of the questionnaire. As shown in Table 2, the standardized factor loadings for the 15 variables are all greater than 0.8, and the Cronbach’s α and CR values are all above 0.7, falling within the acceptable range [56]. Additionally, the Average Variance Extracted (AVE) values are all above 0.5, indicating that the measurement model has good reliability and convergent validity [55].

**Table 2:** Reliability Testing of Various Variables.

Constructs	Items	Standardized Loading	Cronbach’ α	C.R.	AVE
Heritage Image [51]	This cultural heritage site is famous for its long history and reputation.	0.922	0.893	0.934	0.824
	This cultural heritage site has established a good image in the minds of its tourists.	0.88			
	This cultural heritage site reflects its historical colonial atmosphere and cultural blend.	0.921			
Sport Events Image [52]	Toulou Heritage Marathon is a pleasant event.	0.917	0.953	0.964	0.843
	Toulou Heritage Marathon is a novel event.	0.931			
	Information regarding the Toulou Heritage Marathon event is easy to obtain.	0.925			
	The leverage activities such as exhibition, food, cultural and creative products are quite attractive.	0.903			
	I am impressed with the runway and route design of the Toulou Heritage Marathon.	0.915			
Place Attachment [43]	I think coming to Yongding for leisure and travel is part of my life.	0.891	0.908	0.942	0.845
	Yongding infrastructure and environment are the best to me.	0.953			
	If I donot come to Yongding, I would lose my friends.	0.911			
Destination Loyalty [53]	I would come to Yongding, once I have time.	0.885	0.899	0.929	0.767
	I would take the initiative to share with my friends the experience and feelings of Yongding.	0.884			
	I would recommend friends to travel to Yongding.	0.875			
	As long as events held in Yongding, I would definitely come.	0.858			

Furthermore, this study uses the Heterotrait-Monotrait Ratio (HTMT) as a reference indicator. Table 3 shows that all HTMT values are below 0.9, meeting the suggested threshold recommended by Hair et al., indicating that the discriminant validity among the variables in this study is acceptable [55].

**Table 3:** Discriminant Validity (HTMT).

	Destination Loyalty	Heritage Image	Place Attachment	Sport Events Image
Destination Loyalty				
Heritage Image	0.803			
Place Attachment	0.741	0.647		
Sport Events Image	0.443	0.487	0.327	

At the same time, collinearity diagnostics show that the variance inflation factor (VIF) values for the exogenous variables (ranging from 2.066 to 5.064) are all below the critical threshold of 10.0, indicating that the collinearity issue among the data in this study is not severe [57]. Finally, as important indicators for evaluating PLS-SEM, the R-squared values (Destination Loyalty: 0.617; Heritage Image: 0.203; Place Attachment: 0.338) and the predictive relevance (Q^2^) values (Destination Loyalty: 0.469; Heritage Image: 0.158; Place Attachment: 0.286) all meet acceptable levels, suggesting that the model has moderate predictive relevance [43].

### 4.3. Structural equation model testing

The path coefficients and significance effects of the model shown in Table 4. The results showed that Sport Events Image could positively affect Destination Loyalty (β = 0.086, t = 2.04, p < 0.05). Sport Events Image positively affected Heritage Image (β = 0.454, t = 5.092, p < 0.001). Heritage Image positively affected Destination Loyalty (β = 0.469, t = 7.951, p < 0.001). Heritage Image could positively affect Place Attachment (β = 0.561, t = 11.171, p < 0.001). Place Attachment had a significant positive effect on Destination Loyalty (β = 0.368, t = 5.178, p < 0.001). However, it is not difficult for Sport Events Image to positively and significantly affect Place Attachment (β = 0.051, t = 1.044, p > 0.05). The results support hypothesis H1, H2, H3, H6, and H7. Previous studies have indicated that event images affect destination loyalty through factors such as destination image, event satisfaction, and event attachment [53]. The research findings validate the cognitive reconstruction effect of sports event image on heritage image. The Yongding Tulou Marathon, through its visual symbol system (such as the event logo integrating the outline of tulou and the symbols of the construction techniques of Hakka tulou), aesthetically fuses the “dynamic vitality” of sports competition with the “historical weight” of cultural heritage. The timing chips worn by runners during the event are embedded with QR codes containing knowledge about tulou architecture, which triggers the update of heritage cognition simultaneously during the running process, creating a unique experience of “running while learning”. At the same time, it further strengthens the emotional empowerment of heritage image on place attachment, achieving a leap from cognitive identification to emotional commitment. The strong driving effect of heritage image on place attachment reveals the emotional transformation potential of cultural heritage. Tourists’ real perception of the tulou heritage image (such as the well-preserved family living spaces) triggers cognitive resonance, and through the self-categorization theory, transforms cognition into emotional belonging to the destination.

**Table 4:** Structural model of the hypotheses tests.

	ES	S.E.	t	P
H1: Sport Events Image→Destination Loyalty	0.086	0.042	2.04	0.041*
H2: Sport Events Image→Heritage Image	0.454	0.089	5.092	***
H3: Heritage Image→Destination Loyalty	0.469	0.059	7.951	***
H5: Sport Events Image→Place Attachment	0.051	0.049	1.044	0.296
H6: Heritage Image→Place Attachment	0.561	0.05	11.171	***
H7: Place Attachment→Destination Loyalty	0.368	0.071	5.178	***

Note.*p < 0.05,***p < 0.001.

Hypothesis 4, stating no significant impact of event image on place attachment, might be due to the fact that the initial motivation for some tourists to come to Tulou is to participate in sports events. As the Tulou Marathon is held annually as a regular sports event, their enthusiasm for the event itself is significantly higher than their attachment to the destination. Their focus is on the professional aspects of the event, such as its organization, logistics, and course, rather than the specific location where the event is held. Additionally, Yongding, located in the southwestern part of Longyan City, Fujian Province, is not easily accessible. It is suitable for self-driving, and there are no direct high-speed trains to the destination. Moreover, it takes about 3 hours by taking a bus from Xiamen takes about 3 hours, and about 4 hours from Fuzhou. Therefore, the inconvenience of transportation makes it difficult for tourists to form local attachment.

### 4.4. Mediation analysis

Due to the potential presence of multiple mediation effects in the model structure, this study used SmartPLS software for analysis. By performing bootstrap resampling (N = 5000), the results are shown in Table 5. Under a 95% confidence interval, the study found 2 mediation paths in the effect of Sport Events Image on Destination Loyalty.H4: Heritage Image played a mediating role between Sport Events Image and Destination Loyalty (β = 0.213, t = 3.89, p < 0.001). H9: Heritage Image and Place Attachment played a chain mediating role between Sport Events Image and Destination Loyalty (β = 0.094, t = 3.197, p < 0.01).Among them, only H8: Place Attachment plays a mediating role between Sport Events Image and Destination Loyalty (β = 0.019, t = 0.965, p > 0.05),it did not establish a mediating effect. The research results show that no significant difference in the mediating effect of place attachment on destination loyalty, displaying the lack of a significant relationship with tourists’ place attachment. Cultural heritage plays a crucial role in the formation of visitor destination loyalty. Heritage image could better exert its effect on destination loyalty formed during visitor participation in the event, demonstrating a strong mediating effect.Moreover, the cognitive event image formed by tourists during event participation can also influence destination loyalty through the dual mediating effects of place attachment and heritage image.

**Table 5:** Summary of Mediation Tests.

Impact Path	ES	S.E.	t	P
H4: Sport Events Image→Heritage Image→Destination Loyalty	0.213	0.055	3.89	***
H8: Sport Events Image→Place Attachment→Destination Loyalty	0.019	0.02	0.965	0.335
H9: Sport Events Image→Heritage Image→Place Attachment →Destination Loyalty	0.094	0.029	3.197	0.001**

Note.*p < 0.05,***p < 0.001.

The high traffic attribute of sports events is fundamentally in conflict with the deep generation mechanism of local attachment (which requires long-term and multi-dimensional interaction). In the Yongding case, the superimposition of geographical marginality, instrumental motivation and superficial cultural narrative ultimately makes it impossible for the event image to independently activate local attachment, and it is necessary to rely on the cognitive transformation of the heritage image to achieve indirect influence.The reason for this outcome, this study suggests that geographical location of Yongding Tulou is relatively remote, and transportation is not convenient, making it difficult for tourists to form a local attachment to it, let alone destination loyalty. In addition, based on the background information of the data, most participants in the Tulou Marathon are from Fujian province or neighboring provinces (Zhejiang, Jiangxi, Guangdong). Participants from other provinces in China are fewer, not to mention international participants. There are 3 possible reasons for this. Firstly, the sample was collected at post Covid-19 period, in December 2022. Although the international situation has stabilized, and domestic restrictions have been lifted, individuals or groups are still engaged in self-protection due to the shadow of concern and fear. People’s preferences for participation or gatherings have not fully returned to pre-pandemic levels. Secondly, the three neighboring provinces are adjacent to Fujian, and the main reason for participants to choose Tulou to participate in the heritage marathon, besides being attracted by the heritage image, is because of the principle of proximity. Other heritage marathon events, such as Dujiangyan and Dunhuang, are relatively distant. Therefore, for heritage marathon events, it is difficult for the event image to generate destination loyalty solely through tourists’ place attachment. Finally, The cultural narrative of the event is fragmented. The event design fails to achieve an immersive cultural translation (for instance, the LOGO only contains the outline of the earthen building and lacks narrative layers such as family genealogies and rammed earth craftsmanship), resulting in the heritage image remaining at the visual surface. In contrast to the “AR Legacy Unlocking” mechanism of the Nara Marathon, the Yongding event failed to forcibly trigger cognitive deepening through task-based design. Although tourists cannot directly have a significant impact on their loyalty through the mediating role of place attachment, they can achieve a significant impact through the chain mediation effect of heritage image and place attachment. This study suggests that it is difficult for sports events alone to directly influence tourists’attachment to the heritage site. However, once tourists recognize and appreciate the cultural heritage image behind the event site, it gradually shapes their place attachment, thereby leading to stronger loyalty behaviors.

### 4.5. Multi-group analysis

This study uses the multi-group analysis (PLS-MGA) feature of SmartPLS 3.0 software to discuss the moderating effects of demographic characteristics on the research model [19,58]. Previous research indicates that the case difference between categories should not exceed twice the value [49]. Therefore, this study divides the sample into four groups based on gender, age, participation frequency, and monthly running distance for exploration: Group 1: Among the tourists, there are 183 males and 95 females; Group 2: Age below 35 years includes 94 people, and age above 30 years includes 184 people; Group 3: First-time participants are 161, and repeat participants are 117; Group 4: Monthly running distance greater than 50 km includes 153 people, and monthly running distance not exceeding 50 km includes 125 people. The data meet the requirements for multi-group analysis and are consistent with real-world situations, making multi-group analysis feasible. Table 6 shows the path differences in multi-group analysis. The empirical results indicate that the direct relationship between sport events image and destination loyalty is moderated by the demographic variables of gender, age, participation frequency, and monthly running distance; the direct relationship between sport events image and heritage image is moderated by age and monthly running distance; the direct relationship between sport events image and place attachment is moderated by participation frequency; thus, Hypothesis H4 is partially supported, specifically including the following points.

**Table 6:** PLS-MGA.

Impact Path	Gender		Age		Participation frequency		Monthly mileage	
	Male	Female	>35	≤35	1	>1	>50	≤50
H1: Sport Events Image→Destination Loyalty	0.035	0.152*	0.124*	0.047	0.048	0.126*	0.153*	0.043
H2: Sport Events Image→Heritage Image	0.443***	0.475***	0.557***	0.261	0.49***	0.414**	0.626***	0.198
H3: Heritage Image→Destination Loyalty	0.479***	0.453***	0.376***	0.56***	0.532***	0.379***	0.44***	0.475***
H5: Sport Events Image→Place Attachment	0.053	0.036	0.057	0.068	−0.021	0.154*	0.1	−0.004
H6: Heritage Image→Place Attachment	0.568***	0.57***	0.631***	0.403***	0.614***	0.489***	0.585***	0.467***
H7: Place Attachment→Destination Loyalty	0.358***	0.392***	0.432***	0.31***	0.308**	0.467***	0.327**	0.414***

Note: ***p < 0.001,**p < 0.01,*p < 0.05.

#### 4.6.1. Gender.

In terms of gender, the influences of Sport Events Image on Heritage Image, Heritage Image on Destination Loyalty, Heritage Image on Place Attachment, and Place Attachment on Destination Loyalty are significant in both male and female groups. However, the influence of Sport Events Image on Place Attachment is not significant in either group, which aligns with past research, indicating that there is not much difference in its impact based on gender. Furthermore, when it comes to the impact of Sport Events Image on Destination Loyalty, male tourists exhibit a tendency towards negative responses. The underlying reason is that female tourists are more emotional, paying more attention to the surrounding environment of the events, including the marketing strategies of the event and the media portrayal. In other words, female participants focus more on the impressions formed by the sporting events at heritage sites and their surroundings, leading to direct experiences. Conversely, male tourists tend to be more rational, focusing on the underlying values of the events, and superficial event images are insufficient to cultivate their loyalty.

#### 4.6.2. Age.

Regarding age, the influences of Heritage Image on Destination Loyalty, Heritage Image on Place Attachment, and Place Attachment on Destination Loyalty are significant in both groups over and under 35 years old. However, the influence of Sport Events Image on Place Attachment is not significant in either age group, which aligns with the overall pattern, indicating that the impact based on age does not vary greatly. Additionally, Sport Events Image has a significant impact on Destination Loyalty in the group over 35 years old, but not in the group under 35 years old. This is attributed to the fact that young adults aged under 35, especially those born in the 1990s and early 2000s1 have a preference for fashionable and stimulating sports activities with a short, quick pace. Marathons, which are time-consuming and physically demanding, are less attractive to them. In contrast, those over 35 often have stable careers and incomes, showing a preference for more substantial and challenging events like marathons. As a result, they are more willing to engage in travel related to such events and show greater loyalty to them.Moreover, in the path of Sport Events Image influencing Heritage Image, heritage image is also a prominent feature of the host location. For individuals under 35, there is more focus on adventure tourism rather than cultural heritage tourism. Conversely, individuals over 35, who are more likely to have stable jobs and families, are more inclined to participate in cultural heritage tourism and use it as an opportunity to educate their children. Thus, the impact of event characteristics on the heritage image is more significant for this older age group.

#### 4.6.3. Participation frequency.

For participation frequency, the influences of Sport Events Image on Heritage Image, Heritage Image on Destination Loyalty, Heritage Image on Place Attachment, and Place Attachment on Destination Loyalty are significant in both first-time participants and repeat participants of the Tulou Marathon. This aligns with the overall pattern, indicating that the impact based on participation frequency does not vary greatly.Additionally, in the path of Sport Events Image influencing Destination Loyalty, first-time participants in the Tulou Marathon find it difficult to develop loyalty. This is fundamentally due to the fact that marathons are highly challenging sports events. For first-time participants, unfamiliarity with the race route means they focus more on the physical and mental aspects of the race, with less attention given to the cultural significance of the host location. In contrast, repeat participants are more familiar with the race route and tend to focus more on the Tulou culture of the host location, which enhances their attachment to the place due to their passion. Similarly, in the path of Sport Events Image influencing Place Attachment, participants who are passionate about the event develop a stronger attachment to the host location, thereby increasing their sense of place attachment.

#### 4.6.4. Monthly mileage.

Regarding the impact of the Heritage Image on Destination Loyalty, Heritage Image on Place Attachment, and Place Attachment on Destination Loyalty, it is evident that both aspects play a crucial role, regardless of whether tourists run more than 50 KM or less. This aligns with the overall pattern, indicating that the impact based on monthly mileage does not vary greatly. Additionally, in the paths of Sport Events Image influencing Destination Loyalty and Sport Events Image influencing Heritage Image, for the group with a monthly mileage exceeding 50 KM, running has become an essential part of their lives. This group, which loves running, gains a sense of achievement from participating in marathons, thus deepening their loyalty to the local area when major marathon events are held. In contrast, for those with a monthly mileage below 50 KM, participating in a marathon is more of a challenge than a pleasure. As a result, they focus more on personal exercise and breaking through their own limits rather than on the tourism activities related to the event, leading to lower loyalty.

## 5. Conclusion and discussion

### 5.1. Research conclusions

This study aims to realize the combination of sports tourism and heritage tourism, and promote the high-quality development of global tourism. It formulated a theoretical model involving heritage image and place attachment, examining their impact on visitor destination loyalty through empirical research. (1) The image of sports events is an important factor affecting visitor destination loyalty through empirical research. But it is difficult to directly affect tourists to have a sense of place attachment; (2) The image of heritage is more deeply rooted in people’s hearts, which can promote tourists’ loyalty and attachment to heritage sites, and at the same time, attachment can directly stimulate tourists’ loyalty; (3) The image of sports events can stimulate tourists’ image of heritage, thus enhancing their loyalty to heritage sites. At the same time, the image of sports events can stimulate the tourists’ sense of heritage image and place attachment, and ultimately strengthen the loyalty to the heritage sites. (4) Demographic variables such as gender, age, frequency of participation, and monthly running distance are key factors affecting the mechanism through which sport events image influences destination loyalty.

### 5.2. Theoretical contribution

Initially, an elabration of the Heritage Tourism Research Framework: This study explores the role of sports events in heritage tourism, expanding the research framework on the impact of heritage tourism on destination image. It provides theoretical guidance for managing the image of heritage tourism destinations. In heritage marathon events, sports events represent the most direct perception for visitors participating in sports activities at heritage tourism sites. However, previous studies on heritage tourism perception primarily focused on emotional cognition [59], and seldom introduced sports events as a variable in heritage tourism research. This study clarifies the critical role of sports events in heritage tourism, strengthens the unique relationship between heritage tourism and sports tourism in specific contexts, addresses gaps in heritage tourism research, and contributes to destination management and market recovery.

Secondly, Understanding the Impact of Sports Events on Tourist Loyalty: This study analyzes the response patterns of tourist loyalty to sports events and reveals the relationship mechanism between visitor perceptions of cultural heritage sites and the tourism behavior intentions of source market groups. It reexamines the guiding role of destination image on travel behavior intentions. Previous research mostly explored mediating variables such as travel motivation [60] and authenticity [61] from a micro perspective, focusing on how visitor perceptions influence travel intentions and decision-making. This study, combining characteristics of heritage tourism, introduces the special variable of heritage image and reexamines the logical relationship between visitor perceptions of sports events and tourism behavior intentions of source market groups. It helps uncover the “black box” of the relationship between visitor perceptions and needs, providing theoretical insights into the relationship between sports tourism and heritage tourism and expanding the theoretical boundaries and application scope of destination image’s guiding role.

Lastly, differentiated Effects of Tourist Characteristics: This study investigates how different types of tourist characteristics influence behavioral differences, clarifying the causal logic behind these differences. Previous research suggests that demographic characteristics such as age and culture are significant determinants of tourist behavior tendencies [19]. By integrating sports event characteristics and designing demographic variables such as gender, age, frequency of participation, and monthly running distance, the study uses PLS-MGA analysis to identify varying impacts on behavior among different groups. This research highlights the importance and differences of demographic characteristics, providing new theoretical foundations for studying the impact of sports events on tourist loyalty.

### 5.3. Research implications

Based on these conclusions, the following suggestions are proposed to accelerate the integration of the “sports+” ecosystem, create distinctive sports culture IP, enhance visitor destination loyalty, and promote sports as a window for the tourism industry’s development, contributing to the high-quality development of the sports tourism industry.

Leveraging the benefits of heritage image to enhance visitor destination loyalty. Destination managers should capitalize on the unique natural and cultural advantages of World Cultural Heritage to promote visitor recognition of the event venue. This can be achieved by using sports events (projects) as well as a series of supporting leisure and entertainment activities. For example, developing related obstacle-based exports games set in Tulou, Hakka cultural performances, experiential activities for studying heritage, and digital cultural tourism experiences with a martial arts theme can enhance visitor recognition of the heritage image of Yongding Tulou.

Focusing on visitor emotional identification to improve event service systems. Emotional identification is a critical factor influencing tourists’ intentions to revisit or participate in sports events again. A comprehensive and mature service system can not only improve tourists’ attitudes toward the event but also strengthen the psychological connection between tourists and the event, fostering emotional identification. Event organizers should strive to augment the visitor service system, enhance tourists’ emotional service encounters, and nurture a favorable sentiment towards the event. Previous research has shown to to the efficacy of customized emotional marketing tactics aimed at specific demographics in bolstering consumers’ intentions to revisit. For example, to encourage tourists to participate in the event again, event organizers should provide corresponding encouragement and support to repeat participants. Through methods such as giving souvenirs and prioritizing participation qualifications, participants feel the importance of this event and deepen their emotional connection with the event organizers.

Finally, to enhance the appeal and participation of sports events at heritage sites, it is recommended to develop targeted marketing strategies based on visitor characteristics such as gender, age, frequency of participation, and monthly running volume. Firstly, based on gender differences, differentiated activity plans can be designed: male visitors may prefer competitive and challenging events, while female visitors may focus more on experiential and social aspects. Therefore, segmenting the activity formats can cater to the needs of different genders. Secondly, regarding age groups, younger visitors may prefer extreme sports and emerging events, while middle-aged and older visitors may prioritize health and comfort. Developing tiered event arrangements can not only meet the needs of different age groups but also enhance the overall appeal of the events. Additionally, by analyzing visitor participation frequency, it is possible to identify frequent participants and new attendees. Exclusive rewards and discounts can be offered to frequent participants to boost their loyalty and long-term engagement [62]. For new participants, it is essential to design more user-friendly entry-level activities to lower participation barriers and stimulate their interest and enthusiasm. Finally, based on monthly running volume data, tailor event challenges to suit visitors with different running volumes. High-volume runners can participate in high-intensity events to satisfy their craving for challenges, while lower-volume runners can engage in more relaxed and enjoyable events to gradually improve their fitness level and sense of involvement. Through these targeted marketing strategies, we can more effectively attract and retain visitors with different characteristics, enhance the overall participation and satisfaction of sports events at heritage sites, and thereby promote the sustainable development and brand influence of these events.

### 5.4. Limitations and future research

The influencing factors of visitor destination loyalty are diverse, and this study only explored the impact and mechanism of event image in heritage sites conducting sports events. Subsequent research should further explore other factors affecting the mechanism of visitor destination loyalty, providing richer theoretical support for the incubation and advocacy of tourists’ attitudes and behaviors in sports tourism. The study confirmed the partial mediating effects of heritage image and place attachment on visitor destination loyalty. However, it is evident that other influencing factors may exist as chain or parallel mediators between event image and visitor destination loyalty. This requires further validation in future research. Additionally, in interviews with tourists, it was found that the familiarity of event volunteers with the local World Heritage site significantly influenced heritage image. Future research should consider this aspect for further testing.
